# 国人弥漫大B细胞淋巴瘤P53表达的判读标准及预后价值探讨

**DOI:** 10.3760/cma.j.issn.0253-2727.2022.12.006

**Published:** 2022-12

**Authors:** 云飞 时, 子芬 高, 向红 李, 丽改 郭, 勤龙 郑, 孟平 龙, 丽娟 邓, 婷婷 杜, 玲 贾, 炜 赵, 晓昕 宋, 敏 李

**Affiliations:** 1 北京大学肿瘤医院暨北京市肿瘤防治研究所病理科，恶性肿瘤发病机制及转化研究教育部重点实验室，北京 100142 Key Laboratory of Carcinogenesis and Translational Research (Ministry of Education), Department of Pathology, Peking University Cancer Hospital & Institute, Beijing 100142, China; 2 北京大学医学部病理学系/北京大学第三医院病理科，北京 100191 Department of Pathology, Peking University Third Hospital, Beijing 100191, China; 3 北京高博博仁医院病理科，北京 100070 Department of Pathology, Beijing Boren Hospital, Beijing 100070, China; 4 北京高博博仁医院医学检验科分子诊断实验室，北京 100070 Medical Laboratory of Molecular Diagnostic Laboratory, Beijing Boren Hospital Department, Beijing 100070, China; 5 北京大学肿瘤医院暨北京市肿瘤防治研究所淋巴瘤科，恶性肿瘤发病机制及转化研究教育部重点实验室，北京 100142 Key Laboratory of Carcinogenesis and Translational Research (Ministry of Education/Beijing), Department of Lymphoma, Peking University Cancer Hospital & Institute, Beijing 100142, China; 6 首都医科大学临床检验中心，北京 100069 Clinical Laboratory Center, Capital Medical University, Beijing 100069, China; 7 河北省眼科医院病理科，石家庄 054001 Department of Pathology, Hebei Eye Hospital, Shijiazhuang 054001, China

**Keywords:** 弥漫大B细胞淋巴瘤, 肿瘤抑制蛋白质p53, 免疫组织化学, 突变, 预后, Lymphoma, large b-cell, diffuse, Tumor Suppressor Protein p53, Immunohistochemistry, Mutation, Prognosis

## Abstract

**目的:**

探索在国人弥漫大B细胞淋巴瘤（DLBCL）中免疫组织化学染色（IHC）检测P53蛋白表达状态预测TP53基因突变风险的能力，以及P53表达差异的预后评估价值。

**方法:**

收集北京高博博仁医院2021年1月至2021年12月同时进行二代测序（NGS）和IHC检测的DLBCL病例51例，依据P53的IHC染色将P53蛋白表达状态分为缺失（<1％）、弥漫（>80％）和不均一（1％～80％）3组，将缺失及弥漫表达归为TP53突变高风险组；与NGS结果对比分析IHC预测TP53突变风险的敏感性和特异性。收集北京大学肿瘤医院2016年6月至2019年9月有完整随访资料的DLBCL患者131例，制作组织芯片并通过IHC检测P53表达，评估P53表达差异的预后价值。

**结果:**

51例同时行IHC和NGS检测的病例中，TP53突变高风险23例（7例缺失，16例弥漫），NGS证实22例存在TP53突变；TP53突变低风险组28例，仅1例证实有TP53突变。IHC预测TP53突变风险敏感性95.7％，特异性96.4％。NGS共检出26个TP53突变位点，等位基因突变频率为61.57％（13.41％～86.25％），P53弥漫组检出16个错义突变、2个剪切位点突变；缺失组检出6个截短突变、1个剪切位点突变；不均一组检出1个截短突变。纳入预后分析的131例DLBCL患者中，IHC显示29.0％（38/131）为TP53突变高风险（17例弥漫、21例缺失）。多因素分析显示TP53突变高风险（*HR*＝2.612，95％ *CI* 1.145～5.956，*P*＝0.022）是影响总生存的独立危险因素。

**结论:**

IHC检测P53蛋白表达缺失（<1％）或弥漫（>80％）TP53突变风险高，IHC预测TP53突变的敏感性与特异性高，TP53突变高风险是预后不良的独立影响因素。

## Abstract (en)

**Objective:**

To explore the feasibility of predicting TP53 mutation risk by immunohistochemical staining（IHC）pattern of P53 in Chinese diffuse large B-cell lymphoma（DLBCL）and its correlation with a prognostic difference.

**Methods:**

Between January 2021 and December 2021, 51 DLBCL cases at Beijing Boren Hospital were gathered. These cases had both IHC and next-generation sequencing（NGS）results. IHC classified the P53 protein expression pattern into a loss（<1％）, diffuse（>80％）, and heterogeneous（1％-80％）. The sensitivity and specificity of the predicting TP53 mutation by IHC were assessed by comparing the results of the NGS, and the TP53 high mutation risk group included both loss and diffuse expression of P53. From June 2016 to September 2019, Peking University Cancer Hospital collected 131 DLBCL cases with thorough clinicopathological and follow-up data. From their tumor blocks, tissue microarray blocks were made for IHC evaluation of P53 expression pattern, and prognosis effect of P53 studies.

**Results:**

Among 51 cases with both IHC and NGS results, 23 cases were classified as TP53 high mutation risk（7 cases loss and 16 cases diffuse）, 22/23 cases were proved with mutated TP53 by NGS. Only 1 of the 28 cases classified as TP53 low mutation risk was proved with mutated TP53 by NGS. IHC had a sensitivity and specificity of 95.7％ and 96.4％ for predicting TP53 mutation. NGS identified a total of 26 TP53 mutations with a mutation frequency of 61.57％（13.41％–86.25％）. In the diffuse group, 16 missense mutations and 2 splice mutations were detected; 6 truncating mutations and 1 splice mutation were detected in the loss group; 1 truncating mutation was detected in the heterogeneous group. Multivariate analysis demonstrated that TP53 cases with high mutation risk have impartial adverse significance for the 131 patients included in survival analysis（*HR*＝2.612, 95％ *CI* 1.145–5.956, *P*＝0.022）.

**Conclusion:**

IHC of P53 exhibiting loss（<1％）or diffuse（>80％）pattern indicated TP53 high mutation risk, IHC can predict TP53 mutation with high specificity and sensitivity. TP53 high mutation risk is an independent predictor for adverse survival.

弥漫大B细胞淋巴瘤（DLBCL）是最常见的淋巴瘤类型[1]，生物学行为具有强异质性，肿瘤细胞起源（Cell of Origin，COO）分型是广泛认可的病理学预后指标[2]。利妥昔单抗和蒽环类药物的联合应用极大提高了DLBCL的疗效和预后[3]–[5]，但复发难治病例仍高达40％[6]。我们既往对新的病理标志物如双重打击淋巴瘤进行研究[7]。近年TP53基因突变与DLBCL的预后相关性也越来越受到关注[8]，TP53突变在DLBCL中检出率约20％[8]，并在DLBCL分子分型中作为A53亚型分型的重要依据，具有一定的预后和治疗提示价值[9]。目前在卵巢癌等肿瘤中可通过免疫组织化学染色（IHC）显示P53表达状态（>80％为弥漫过表达，<1％为表达缺失）以提示TP53基因突变状态已得到较为广泛的认可[10]–[11]。尽管DLBCL中也存在P53蛋白“过表达”或“表达缺失”[12]，但上述判断标准是否适用DLBCL尚不明确，部分研究以P53表达比例≥50％预测TP53突变，未涵盖P53表达缺失，亦未获得一致结论[3],[6],[8],[13]–[14]。

因此，本研究收集了部分同时行TP53二代测序（NGS）和P53 IHC检测的病例，探索在DLBCL中IHC检测P53表达状态预测TP53基因突变状态的病理学评估标准及其可行性。并在此基础上收集了一组临床、病理及随访信息完整的初治DLBCL病例，分析P53表达差异对预后的影响，期待为国人DLBCL中P53评估提供一些依据。

## 病例与方法

一、病例资料

1. 收集2021年1月至2021年12月间北京高博博仁医院同时进行P53的IHC及靶向外显子（包含TP53）NGS检测的51例DLBCL患者，进行诊断试验。

2. 收集北京大学肿瘤医院2016年6月至2019年9月间确诊的131例初治DLBCL患者，进行预后分析。纳入标准：（1）具有完整的临床和治疗信息；（2）存档组织标本符合制作组织芯片的要求。

所有病例依据2016年WHO淋巴造血系统肿瘤分类标准[1]，经2名以上血液病理学专家复核诊断。

二、组织芯片制作

HE切片勾选石蜡标本肿瘤细胞丰富区域，使用法国Alphelys公司组织芯片仪制作组织芯片（每例均含2个直径1 mm的组织条，使用芯片仪自带程序标记对应位置）。

三、免疫组织化学染色及其结果判读

应用瑞士Roche公司BENCHMARK-XT自动免疫组织化学染色机完成，Ⅰ抗BCL2、BCL6、CD10、CD20、Mum1、P53均购自美国安捷伦DAKO公司；CD3_ε_、C-MYC、Ki67均购自北京中杉金桥生物技术有限公司，DAB显色。P53阳性对照使用卵巢癌阳性组织，其余为反应性增生的淋巴组织。阴性对照均为Ⅰ抗稀释液代替Ⅰ抗进行染色。

仅计数明确着色的肿瘤细胞在所有肿瘤细胞中占比。CD10、BCL6、MUM1≥30％为阳性[2]。BCL2≥50％为高表达，MYC≥40％为高表达[15]。P53表达状态分为缺失（<1％）、弥漫（>80％）以及不均一（1％～80％）（图1），缺失和弥漫表达归为TP53突变高风险组[10]–[11]。同时便于对比按P53是否≥50％分为高表达或低表达组[6]。

**图1:**
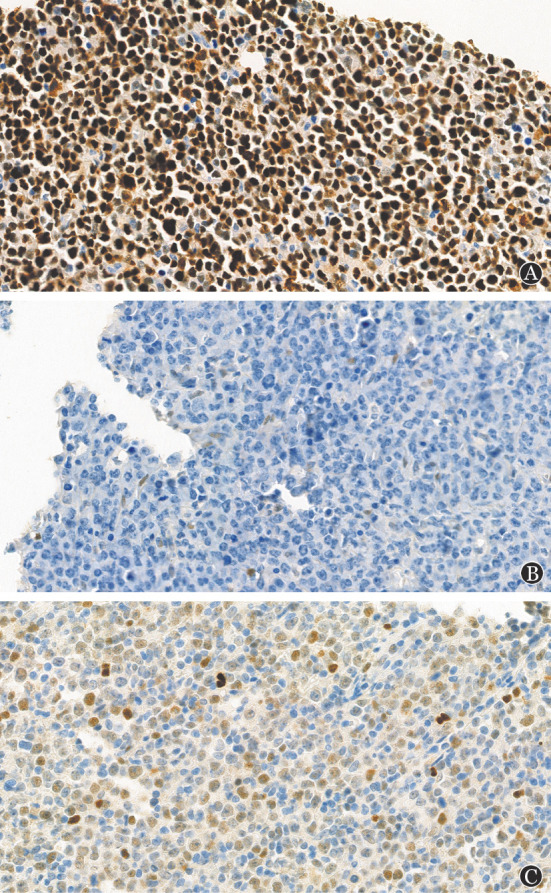
弥漫大B细胞淋巴瘤P53表达状态（免疫组化染色，UltraView法，高倍）A：P53弥漫表达（>80％强阳性）；B：P53缺失（<1％），非瘤细胞偶表达；C：P53不均一表达（1％～80％）

四、NGS

使用GeneRead DNA FFPE Kit（Qiagen公司，德国）分离提取FFPE样品DNA，进行定性定量检测。使用KAPA hyper plus试剂盒利用酶切方法完成预文库构建，预文库与定制的Kapa Hyper choice panel（NimbleGen，美国）进行杂交捕获，并对目标DNA片段进行富集扩增以生成gDNA文库。使用NextSeq 550测序系统（Illumina，美国）进行高通量测序（深度约2 500×）。使用BWA（0.7.15版）进行参考基因组比对，GATK（4.0.4版）和VarDict（2014版）软件检测单核苷酸变异和小片段插入缺失等，最终使用IGV浏览器人工审核，记录TP53突变。

五、疗效评价与随访

参照2014年淋巴瘤Lugano分期及评效标准进行疗效评价。随访截止日期为2022年1月，总生存（OS）时间定义为从确诊至出现死亡或末次随访的时间间隔。无事件生存（EFS）时间定义为研究病例从确诊直至疾病出现进展、复发、死亡或末次随访的时间间隔。

六、统计学处理

本研究为回顾性研究，应用SPSS 22.0软件进行统计学分析。采用受试者工作特征曲线（ROC曲线）评价IHC预测TP53突变的敏感性及特异性。采用Cox等比例风险回归模型进行影响OS和EFS的单因素及多因素分析，单因素分析中*P*<0.1的变量纳入多因素分析，采用逐步法对纳入多因素模型的变量进行筛选并确定最终的危险因素。*P*<0.05为差异有统计学意义。

## 结果

一、P53表达状态预测TP53突变风险

51例同时行IHC和NGS检测的病例中，TP53突变高风险组23例（弥漫组16例、缺失组7例），其中22例NGS检测存在TP53突变，仅1例弥漫组NGS未检出TP53突变；28例TP53突变低风险组，仅1例NGS检出TP53突变。以NGS检出TP53突变为“金标准”，使用IHC突变高风险与低风险分组预测TP53突变风险敏感性是95.7％，特异性是96.4％；按P53≥50％阈值进行分组，预测TP53突变风险敏感性仅65.2％，特异性仅21.4％。

本组病例NGS共检出26个TP53突变位点，中位等位基因突变频率为61.57％（13.41％～86.25％），大部分突变（92.3％，24/26）分布在TP53 DNA结合域。P53弥漫组检出16个错义突变，2个剪切位点突变；缺失组检出6个截短突变，1个剪切位点突变；不均一组检出1个截短突变（图2）。

**图2:**
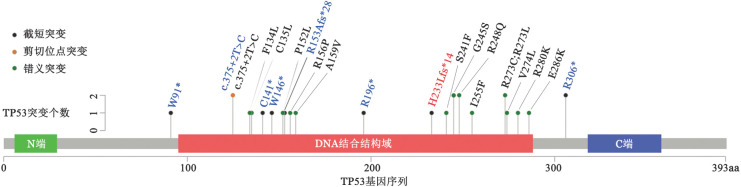
51例弥漫性大B细胞淋巴瘤患者二代测序检出TP53突变位点示意图 注 黑字为P53弥漫（>80％）组突变位点，蓝字为P53缺失（<1％）组突变位点，红字为P53不均一（1％~80％）组突变位点，*示终止密码子

二、131例有完整资料DLBCL患者P53表达情况及预后分析

1. 一般情况：纳入131例北京大学肿瘤医院有完整的临床及随访信息的DLBCL患者，中位年龄56（24～84）岁，其中>60岁44例（33.6％）；男77例（58.8％）；Ann Arbor Ⅲ～Ⅳ期67例（51.1％）；有B症状（发热、盗汗、体重减轻）50例（38.2％）；ECOG>1分10例（7.6％）；30例（22.9％）结外侵犯部位>1个；68例（51.9％）血清LDH水平升高（>240 U/L）；出现大包块者40例（30.5％）；IPI 3～5分36例（27.5％）。同时出现MYC及BCL2蛋白双高表达（DEL）的共22例（16.8％）。全部病例均接受了CHOP方案（环磷酰胺+阿霉素+长春新碱+泼尼松）为主的标准化一线治疗，其中79例（60.3％）患者联合了利妥昔单抗治疗。疗效评估87例（66.4％）完全缓解（CR），17例（13.0％）部分缓解（PR），26例（19.8％）疾病进展（PD），1例（0.8％）未评价疗效。患者5年OS及EFS率分别为85.7％、52.5％。

2. IHC检测P53表达情况：131例患者中P53中位表达水平为17.5％（0～95.0％），其中弥漫组17例（13.0％）、缺失组21例（16.0％）、不均一组93例（71.0％），38例（29.0％）归为TP53突变高风险组。

3. 预后因素分析：单因素分析结果详见表1，Ann Arbor Ⅲ～Ⅳ期（*P*＝0.005）、结外侵犯>1处（*P*＝0.011）、血清LDH>240 U/L（*P*＝0.053）、IPI 3～5分（*P*＝0.005）、DEL（*P*＝0.051）、TP53突变高风险（*P*＝0.081）在0.1的水平上是影响OS的危险因素；Ann Arbor Ⅲ～Ⅳ期（*P*<0.001）、结外侵犯>1处（*P*<0.001）、血清LDH>240 U/L（*P*＝0.018）、IPI 3～5分（*P*<0.001）、一线治疗未用利妥昔单抗（*P*＝0.009）、DEL（*P*＝0.010）是影响EFS的危险因素。将上述变量纳入多因素COX模型，通过逐步法进行变量筛选，最终结果显示Ann Arbor Ⅲ～Ⅳ期（*HR*＝4.532，95％ *CI* 1.659～12.381，*P*＝0.003）、DEL（*HR*＝2.528，95％ *CI* 0.968～6.604，*P*＝0.058）及TP53突变高风险（*HR*＝2.612，95％ *CI* 1.145～5.956，*P*＝0.022）是影响OS的独立危险因素。Ann Arbor Ⅲ～Ⅳ期（*HR*＝3.236，95％ *CI* 1.761～5.948，*P*<0.001）、DEL（*HR*＝2.012，95％ *CI* 1.082～3.741，*P*＝0.027）、血清LDH>240 U/L（*HR*＝1.671，95％ *CI* 0.920～3.037，*P*＝0.092）及一线治疗未用利妥昔单抗（*HR*＝2.799，95％ *CI* 1.615～4.851，*P*<0.001）是影响EFS的独立危险因素。

**表1:** 影响131例初治弥漫大B细胞淋巴瘤患者总生存（OS）及无事件生存（EFS）的单因素分析

因素	OS			EFS		
	*HR*	95% *CI*	*P*值	*HR*	95% *CI*	*P*值
年龄>60岁	1.861	0.819~4.226	0.138	1.475	0.871~2.498	0.148
女性	0.896	0.387~2.072	0.797	0.844	0.497~1.435	0.531
Ann Arbor Ⅲ~Ⅳ期	4.184	1.550~11.291	0.005	3.296	1.850~5.870	<0.001
B症状	1.748	0.770~3.965	0.182	1.306	0.774~2.204	0.317
ECOG>1分	2.663	0.906~7.832	0.075	1.315	0.525~3.294	0.559
结外侵犯>1处	3.013	1.294~7.012	0.011	2.622	1.530~4.494	<0.001
血清LDH>240 U/L	2.416	0.989~5.902	0.053	1.915	1.119~3.280	0.018
有大包块	1.628	0.704~3.766	0.255	1.237	0.714~2.143	0.448
IPI 3~5分	3.303	1.445~7.552	0.005	2.643	1.561~4.474	<0.001
一线治疗未用利妥昔单抗	1.742	0.768~3.952	0.184	1.991	1.188~3.336	0.009
DEL	2.566	0.996~6.615	0.051	2.229	1.212~4.098	0.010
non-GCB型	1.107	0.469~2.614	0.816	1.519	0.862~2.675	0.148
TP53突变高风险	2.083	0.913~4.752	0.081	1.246	0.719~2.160	0.434
P53表达≥50%	1.259	0.463~3.424	0.652	1.174	0.619~2.226	0.623
Ki-67>80%	1.006	0.424~2.386	0.990	1.053	0.621~1.784	0.848

注 ECOG：美国东部肿瘤协作组体能状态评分；LDH：乳酸脱氢酶；IPI：国际预后指数；DEL：MYC与BCL2蛋白双表达淋巴瘤；non-GCB型：非生发中心型；TP53突变高风险：免疫组化显示P53弥漫（>80％）或缺失（<1％）

## 讨论

TP53基因是最早发现的抑癌基因之一，编码的P53蛋白可通过诱导细胞周期停滞、诱发细胞凋亡及修复DNA损伤等机制减少遗传不稳定性，在超半数实体瘤中存在TP53突变导致P53功能失活并提示预后不良，淋巴瘤中TP53突变发生率仅12.5％，在DLBCL中约20％，尽管发生率相对低，TP53突变在DLBCL的发生、进展及耐药中仍具有重要意义[8],[16]–[17]。

由于TP53编码基因较长，突变位点具有不确定性，传统的Sanger测序可能会漏检，NGS检测虽更为准确，但费用高、耗时长且涉及生信分析，不易在日常工作中普及。研究发现TP53错义突变通常会导致突变的P53蛋白抗降解而在细胞核中积累，从而出现P53蛋白弥漫表达；提前终止突变和剪接位点突变则导致细胞中P53蛋白表达缺失[10]。因此使得IHC染色检测P53表达状态进而预测TP53基因突变状态成为可能，在卵巢癌和子宫内膜癌中IHC染色预测基因突变状态，具有100％的特异性和96％的敏感性[10]–[11]。但既往研究在DLBCL中通过IHC预测TP53突变及预后通常以50％为截断值，不同研究结论存在差异[6],[12]–[14],[17]。究其原因首先是采用50％截断值，将P53表达缺失纳入低风险组可能造成数据的偏差；其次研究大多使用一代测序检测TP53突变，可能存在漏检；同时IHC染色可能使用手工染色或者不同的自动化染色系统，标准化及可重复性较差；再加上文献中对病理医师判读P53染色强度没有明确要求，加重了判读结果不一致性。我们尝试参照妇科肿瘤中的标准，将P53表达弥漫（比例>80％）和缺失（<1％）作为提示TP53突变高风险的标准模式。初步证实IHC在DLBCL中亦可以较好地预测TP53突变风险，敏感性和特异性均>95％，接近妇科肿瘤研究结果；同时结果显示P53弥漫组绝大部分属于错义突变；P53缺失组绝大部分属于截短突变，突变类型与蛋白表达相关性也基本一致[8],[10]。个别不一致的病例可能受到如IHC和NGS检测方法敏感性、肿瘤转录后调控等影响[8]。因此采取与妇科肿瘤一致以肿瘤细胞>80％强表达为截断值，可以更好地提高阅片的一致性。

单因素生存分析证实Ann Arbor Ⅲ～Ⅳ期、结外侵犯、LDH升高、IPI 3～5分及伴有DEL均为影响OS和EFS的危险因素，与其他文献报道基本一致[15],[18]–[19]。本组病例绝大部分治疗方案含利妥昔单抗，导致COO分型失去预测价值[4]。多因素分析与既往报道一致[18],[20]，进一步证实伴有DEL和Ann Arbor Ⅲ～Ⅳ期是影响国人DLBCL的OS和EFS的独立危险因素，TP53突变高风险是OS独立危险因素，利妥昔单抗时代DEL是比COO分型更强的病理预后指标[15],[18]。

另一个有意义的发现是通过NGS和IHC结果对比进一步确认了DLBCL中TP53突变会导致P53表达缺失（<1％）；我们还初步证实P53表达缺失组预后较差，因此本研究进一步丰富和完善了DLBCL中IHC预测TP53突变和预后的研究数据和结论。

总之，本研究结果证实P53的IHC染色表达弥漫（>80％）和缺失（<1％）可以较为准确预测DLBCL的TP53突变高风险，且TP53突变高风险是OS的独立危险因素。本研究使用的P53 IHC判读标准具有一定的可行性，有利于针对性发现TP53突变、评估预后及开展靶向治疗[9],[21]，最终改善生存。由于本研究为回顾性研究，样本量有限且DEL病例数较低，结论有待进一步前瞻性、大样本研究验证。

## References

[1] Swerdlow SH, Campo E, Harris NL (2017). WHO classification of Tumours of Haematopoietic and Lymphoid Tissues[M].

[2] Hans CP, Weisenburger DD, Greiner TC (2004). Confirmation of the molecular classification of diffuse large B-cell lymphoma by immunohistochemistry using a tissue microarray[J]. Blood.

[3] Liu W, Ji X, Song Y (2020). Improving survival of 3760 patients with lymphoma: Experience of an academic center over two decades[J]. Cancer Med.

[4] Castillo JJ, Beltran BE, Song MK (2012). The Hans algorithm is not prognostic in patients with diffuse large B-cell lymphoma treated with R-CHOP[J]. Leuk Res.

[5] 李 敏, 刘 翠苓, 尹 文娟 (2012). 中国人弥漫大B细胞淋巴瘤新分类模型的预后分析[J]. 中华血液学杂志.

[6] Wang XJ, Medeiros LJ, Bueso-Ramos CE (2017). P53 expression correlates with poorer survival and augments the negative prognostic effect of MYC rearrangement, expression or concurrent MYC/BCL2 expression in diffuse large B-cell lymphoma[J]. Mod Pathol.

[7] 李 敏, 张 秋露, 赵 炜 (2021). 伴MYC、BCL2和(或)BCL6重排的高级别B细胞淋巴瘤在弥漫大B细胞淋巴瘤中的发生率[J]. 中华血液学杂志.

[8] Lu TX, Young KH, Xu W (2016). TP53 dysfunction in diffuse large B-cell lymphoma[J]. Crit Rev Oncol Hematol.

[9] Wright GW, Huang DW, Phelan JD (2020). A Probabilistic Classification Tool for Genetic Subtypes of Diffuse Large B Cell Lymphoma with Therapeutic Implications[J]. Cancer Cell.

[10] Köbel M, Piskorz AM, Lee S (2016). Optimized p53 immunohistochemistry is an accurate predictor of TP53 mutation in ovarian carcinoma[J]. J Pathol Clin Res.

[11] Singh N, Piskorz AM, Bosse T (2020). p53 immunohistochemistry is an accurate surrogate for TP53 mutational analysis in endometrial carcinoma biopsies[J]. J Pathol.

[12] Koduru PR, Raju K, Vadmal V (1997). Correlation between mutation in P53, p53 expression, cytogenetics, histologic type, and survival in patients with B-cell non-Hodgkin's lymphoma[J]. Blood.

[13] Xu-Monette ZY, Wu L, Visco C (2012). Mutational profile and prognostic significance of TP53 in diffuse large B-cell lymphoma patients treated with R-CHOP: report from an International DLBCL Rituximab-CHOP Consortium Program Study[J]. Blood.

[14] 黄 浦, 陈 舒, 杨 鑫 (2019). P53及BCL2蛋白在双表达弥漫大B细胞淋巴瘤中的预后判断价值[J]. 中华血液学杂志.

[15] Staiger AM, Ziepert M, Horn H (2017). Clinical Impact of the Cell-of-Origin Classification and the MYC/BCL2 Dual Expresser Status in Diffuse Large B-Cell Lymphoma Treated Within Prospective Clinical Trials of the German High-Grade Non-Hodgkin's Lymphoma Study Group[J]. J Clin Oncol.

[16] Xu-Monette ZY, Medeiros LJ, Li Y (2012). Dysfunction of the TP53 tumor suppressor gene in lymphoid malignancies[J]. Blood.

[17] Cheung KJ, Horsman DE, Gascoyne RD (2009). The significance of TP53 in lymphoid malignancies: mutation prevalence, regulation, prognostic impact and potential as a therapeutic target[J]. Br J Haematol.

[18] Ma Z, Niu J, Cao Y (2020). Clinical significance of ‘double-hit’ and ‘double-expression’ lymphomas[J]. J Clin Pathol.

[19] Perry AM, Alvarado-Bernal Y, Laurini JA (2014). MYC and BCL2 protein expression predicts survival in patients with diffuse large B-cell lymphoma treated with rituximab[J]. Br J Haematol.

[20] Shi Y, Deng L, Song Y (2018). CD3+/CD8+ T-cell density and tumoral PD-L1 predict survival irrespective of rituximab treatment in Chinese diffuse large B-cell lymphoma patients[J]. Int J Hematol.

[21] Monti S, Chapuy B, Takeyama K (2012). Integrative analysis reveals an outcome-associated and targetable pattern of p53 and cell cycle deregulation in diffuse large B cell lymphoma[J]. Cancer Cell.

